# Microcystin-LR-Triggered Neuronal Toxicity in Whitefish Does Not Involve MiR124-3p

**DOI:** 10.1007/s12640-018-9920-4

**Published:** 2018-06-07

**Authors:** Maciej Florczyk, Paweł Brzuzan, Alicja Łakomiak, Ewa Jakimiuk, Maciej Woźny

**Affiliations:** 1 Department of Environmental Biotechnology, Faculty of Environmental Sciences, University of Warmia and Mazury in Olsztyn, ul. Słoneczna 45G, 10-709 Olsztyn, Poland; 2 Division of Veterinary Prevention and Feed Hygiene, Faculty of Veterinary Medicine, University of Warmia and Mazury in Olsztyn, ul. Oczapowskiego 13, 10-950 Olsztyn, Poland

**Keywords:** microRNA, Neurotoxicity, Gliosis, Circulating microRNA

## Abstract

Microcystin-LR (MC-LR) is a potent hepatotoxin that has also been pointed out of causing neurotoxicity, but the exact mechanisms of action still remain ambiguous and need to be elucidated. Data from studies on mammals show that pathology of astrocyte cells points to perturbations of microRNA signaling. Glial fibrillary acidic protein (GFAP), a neuronal cell/astrocyte-specific protein, and a microRNA-124-3p (MiR124-3p) are among putative triggers and regulators of neuronal cell/astrocyte reactivity. In the present study on whitefish (*Coregonus lavaretus*), we found that *gfap* mRNA contains a putative target site for MIR124-3p, to potentially affect its expression changes. qPCR expression study of *gfap*:MiR124-3p pair in the midbrain of juvenile whitefish, during 28 days of exposure to a repeated subacute dose of MC-LR (100 μg kg^−1^ body mass), showed marginally significant up-regulation of *gfap* only on the 7th day of exposure period which suggests neuronal toxicity. During the whole exposure period, neither midbrain nor blood plasma levels of MiR124-3p were changed. Furthermore, double luciferase gene reporter assay confirmed the lack of MiR124-3p involvement in mediating control over *gfap* mRNA expression. These data show that, although MC-LR may trigger neuronal toxicity in whitefish, this does not involve MiR124-3p in response to the treatment.

**Electronic supplementary material:**

The online version of this article (10.1007/s12640-018-9920-4) contains supplementary material, which is available to authorized users.

## Introduction

Microcystins (MCs) are a challenging group of cyclic heptapeptide hepatotoxins for which a substantial gap in knowledge persists regarding the underlying molecular mechanisms of organ toxicity and injury. Microcystin-LR (MC-LR) is a commonly acting potent environmental agent that has gained a role in causing neurotoxicity. The adverse effects entailed by MC-LR exposure include both behavioral changes, such as different swimming habits and uncommon daily activity (Baganz et al. 1998, 2004; Cazenave et al. 2008), and altered brain physiology (i.e., altered levels of proteins involved in the cytoskeleton assemblage, dysregulated signal transduction, protein degradation, metabolism, transport, apoptosis, and translation) (Wang et al. 2010). In mammals, damage to the central nervous system (CNS) is reflected by increased expression of glial fibrillary acidic protein (GFAP). GFAP/Gfap is an intermediate filament protein expressed by numerous cells in the CNS, including mature astrocytes (Middeldorp and Hol 2011). Following various kinds of injury to the CNS, including chemical insult, astrocytes become reactive and respond in a typical manner termed astrogliosis (Sofroniew and Vinters 2010). Astrogliosis is characterized by rapid synthesis of *gfap* resulting in the formation of a glial scar, a common marker of damage to the CNS (Brenner 2014).

Although the exact mechanisms of action still remain ambiguous, the range of observed neuronal defects points to perturbations of epigenetic factors such as microRNA (miRNA) signaling. MiRNAs are small (19–23 nucleotides), single-stranded non-coding RNAs that act as post-transcriptional regulators of gene expression (Moxon et al. 2008). They negatively modulate up to 60% of mammalian protein-coding genes by interacting with response elements in the 3′ untranslated regions (3′UTRs) of mRNAs and target the mRNAs for degradation and/or inhibit their translation (Bartel 2004; Friedman et al. 2009). Our previous studies on MC-LR effects on fish miRnome have revealed that MC-LR was capable of modulating expression of several microRNAs, members of RNA interference system, in the liver of challenged fish (Brzuzan et al. 2012, 2016; Łakomiak et al. 2016), which points out their mechanistic involvement in the toxicity mechanism. Since molecular background of MC-LR toxicity seems to be similar in different tissues, it was reasonable to assume that similar effects will emerge in the brain. MiRNA profiling of experimental stroke brains has shown alterations in many individual miRNAs, and bioinformatics tools revealed putative functional miRNA:mRNA pairs (Saul et al. 2014). The observation that miRNA levels in the nervous system were changed by MC-LR (Saul et al. 2014) prompted us to investigate the role of fish microRNAs in the context of brain-specific MC-LR toxicity. Moreover, recent studies have shown that disrupted expression of GFAP is associated with altered levels of miRNAs such as MiR125b (Pogue et al. 2010) and MiR145 (Wang et al. 2015). MiR3099 has been shown to target GFAP in the mouse brain (Abidin et al. 2017).

The possibility that MiR124 is able to induce proliferation of reactive astrocytes has recently been demonstrated in mice (Hamzei Taj et al. 2016). MiR124 is the most abundant miRNA in the nervous system of mammals and fish, and it is considered as a nervous system-specific miRNA (Kapsimali et al. 2007; Mishima et al. 2007). Noteworthy, the MIR124 family members were detected in 46 animal species, from *Caenorhabditis* to *Homo sapiens* (Guo et al. 2009). Their abundance in embryonic and adult cortical tissues of various mammalian species range from 5 to 48% of all miRNAs expressed, suggesting that it has a key function in the CNS (Lagos-Quintana et al. 2002; Landgraf et al. 2007). MiR124 was proven to regulate brain development, as well as it is enriched in mature brain tissue, where it plays important roles in neuronal regulation. MiR124 has been shown to be down-regulated in CNS pathologies, such as glioma and medulloblastoma, suggesting its possible involvement in brain tumor progression. On the other hand, MiR124 was found to increase neuronal survival and functional improvement of the neurological deficits (Hamzei Taj et al. 2016) and enhance brain repair in Parkinson’s disease (Saraiva et al. 2016). Mammals treated with MiR124 developed a significantly reduced glial scar area after occlusion of the right middle cerebral artery (Doeppner et al. 2013). More recently, it has been shown that MIR124-3p target sequence in mediated gene transfer restored astrocyte activity in rats, thus showing its utility in studying the miRNA contribution to physiological and pathophysiological processes in the brain (Taschenberger et al. 2017).

Since the above studies link changes in brain GFAP and miRNA expression in mammals, the goals of this study were to dissect involvement of this miRNA:mRNA pair in fish exposed to known neurotoxic environmental agent microcystin-LR (Li et al. 2014). First, we were curious whether there is a functional relationship between expression of MiR124-3p and *gfap* mRNA in vitro, thereby suggesting the possibility of interaction and involvement of this miRNA-mRNA pair in vivo in response to neuronal cells to stressing conditions. To address this issue, we determined nucleotide sequence of the whitefish *gfap* mRNA. Then, using computational approach, we revealed that the 3′UTR of whitefish *gfap* mRNA contains one putative MiR124-3p response element. Secondly, we investigated whether long-term exposure of whitefish to MC-LR would cause alterations in the expression of either molecule in brains similar to those observed in mammals. To this end, we profiled expression levels of MiR124-3p and *gfap* mRNA in the brains of whitefish treated for 1/3, 1, 2, 7, 14, and 28 days with a subacute dose of MC-LR. Additionally, we examined whether plasma levels of MiR124-3p could relate to disrupted *gfap* expression in the brain and possibly serve as a biomarker of MC-LR exposure. Finally, to confirm the functional involvement of MiR124-3p:*gfap* pair, a dual-luciferase reporter assay was performed.

## Material and Methods

### Determining *gfap* cDNA Sequence of European Whitefish

We first obtained a partial *gfap* cDNA sequence of European whitefish, using a designed set of primers (Cla-gfap-F1, Cla-gfap-R1; Supplementary File 1) based on the *S. salar* EST sequence available at GenBank (accession no. GE794087.1). Initial PCR amplification of a starting DNA sequence was conducted using Phusion Flash PCR Master Mix (Thermo Scientific) with the following cycling conditions: 98 °C for 10 s; 30 cycles at 98 °C for 1 s, 60 °C for 5 s, 72 °C for 10 s; followed by 72 °C for 1 min. Following electrophoresis, a band observed at the expected size was cut out and purified using the PureLink™ Quick Gel Extraction and PCR Purification Combo Kit (Invitrogen). The putative cDNA fragment of *gfap* mRNA was then sequenced under contract (Genomed).

The partial *gfap* cDNA sequence was subsequently used as a query in a BLAST search against the sequence deposited in sequence read archive (SRA), accession no. SRX465095, obtained from three pooled normal embryos of lake whitefish (*Coregonus clupeaformis*). The revealed sequence of putative *gfap* of lake whitefish was used as a template to design other sets of primers: Cla-gfap-2-F2 and Cla-gfap-2-R2, Cla-gfap-2-F3 and Cla-gfap-2-R3, and Cla-gfap-2-F4 and Cla-gfap-2-R4 (Supplementary File 1). These primers were used to amplify the partial 5′UTR, entire coding sequence, and partial 3′UTR of European whitefish *gfap*, respectively. PCR was carried out in a final volume of 25 μL as follows: 1 μL of cDNA as template, 12.5 μL of 2 × DreamTaq Green PCR Master Mix (Thermo Scientific), 0.5 μM of forward and reverse primers (Cla-gfap-2-F2 and Cla-gfap-2-R2, or Cla-gfap-2-F3 and Cla-gfap-2-F3, or Cla-gfap-2-F4 and Cla-gfap-2-R4), and 9.5 μL of nuclease-free water. The PCR conditions were 1 cycle at 95 °C for 3 min followed by 30 cycles at 95 °C for 30 s, optimal annealing temperature (as indicated in Supplementary File 1) for 30 s and 72 °C for 1 min. Final extension was carried out at 72 °C for 30 min. The PCR products were cloned using the InsTAclone PCR Cloning Kit (Thermo Scientific) according to the manufacturer’s protocol, with some modifications. PCR amplicons were ligated into pTZ57R/T vector, afterwards the ligation mixtures were transformed into the JM109 competent *Escherichia coli* cells (Promega, USA) using a heat shock (42 °C) transformation method. The resulting transformants were spread on LB agar plates containing X-gal (40 μg/mL), IPTG (40 μg/mL), and ampicillin (100 μg/mL) and incubated overnight at 37 °C. Insert-positive colonies were picked and inoculated into 4 mL of LB with 100 μg/mL of ampicillin. After incubation at 37 °C for 16 h, the plasmids DNA were isolated using a Plasmid Mini Kit (A&A Biotechnology) and sequenced (Genomed). Nucleotide sequences were then assembled using Clustal X 2.1 software into a cDNA sequence of 2160-bp length. This allowed further MiR124-3p target site identification with Segal Lab software.

The obtained *gfap* cDNA sequence was further analyzed by RegRNA 2.0 web server (Chang et al. 2013) to identify the homology of functional RNA motifs and sites, and to align with *gfap* transcript of zebrafish (Ensembl accession no. ENSDART00000028270.6) to predict potential exon–exon junction. In order to find the open reading frame and to acquire the amino acid sequence, cDNA sequence was subjected to the ORF finder software (Wheeler et al. 2004). To identify the domain organization of deduced Gfap protein sequence, the InterPro v. 53 (Mitchell et al. 2015), SMART (Letunic et al. 2015), and Conserved Domains Search (Marchler-Bauer et al. 2015) were used. The molecular weight (MW) and isoelectric point (pI) were determined using the Compute pI/Mw tool (Gasteiger et al. 2005). The sequence of whitefish *gfap* has been deposited in GenBank under accession no. MG182670.

### Phylogenetic Analysis of Whitefish Gfap

To gain insight into the evolutionary relationship of Gfap, the deduced amino acid sequence was compared with those of other vertebrates deposited in NCBI database: XP_02141413 (*O. mykiss*), XP_01404923 (*S. salar*), AAH68410.1 (*D. rerio*), AAB22581.1 (*H. sapiens*), and P03995.4 (*M. musculus*). The sequences were aligned using Clustal W (Larkin et al. 2007) and the output was visualized using BoxShade 3.21 software (www.ch.embnet.org/software/BOX_ form.html). The sequences were also compared using Clustal X 2.1 software to create the percent identity matrix. The phylogenetic tree was constructed using the neighbor-joining method implemented in MEGA 6.06 software (Tamura et al. 2013) with default settings and 1000 bootstrap replicates. The tree was rooted with CED-9 sequences from *Caenorhabditis elegans* (GenBank accession no. NP_499284).

### Construction of Luciferase Reporter Plasmids for Luciferase Assay

To investigate whether MiR124-3p can regulate the expression of whitefish *gfap*, three types of luciferase reporter plasmids were prepared based on the pmirGLO Dual-Luciferase miRNA Target Expression Vector (Promega). To construct the gfap-3′UTR-wt plasmid, the entire 3′UTR region of whitefish gfap, obtained in this study, which contains putative MiR124-3p response element(s), was amplified by PCR using 1 μL of cDNA template, 12.5 μL of 2× Phusion Flash PCR Master Mix (Thermo Scientific), 0.5 μM of gfap-3′UTR-F-DraI and gfap-3′UTR-R-XbaI primers, and 9.5 μL of nuclease-free water. Cycling conditions were as follows: 98 °C for 10 s; 30 cycles at 98 °C for 1 s, 62 °C for 5 s, and 72 °C for 25 s; followed by 72 °C for 2 min. The gel-purified (Invitrogen) product was double digested with *XbaI* and *DraI* restriction enzymes (Thermo Scientific) and cloned into a multiple cloning site of the pmirGLO downstream of the firefly luciferase gene (gfap-3′UTR-wt). Additionally, plasmids containing mutations in the sequence complementary to the seed region of *gfap* (gfap-3′UTR-mut, positive control) were also generated. The resulting plasmids were used to transform the competent *E. coli* JM109 cells (Promega) via the heat shock method. All subsequent steps up to isolation of plasmids from the liquid bacterial culture were carried out as described in the “Determining *gfap* cDNA sequence of European whitefish” section. All plasmids were confirmed by sequencing (Genomed). Sequences of each oligonucleotides used for generation of luciferase reporter plasmids are provided in Supplementary File 1.

### Cell Culture

Human Embryonic Kidney 293T (HEK-293T) cells were purchased from the American Type Culture Collection (ATCC, USA) and maintained in Dulbecco’s Modified Eagle’s Medium/Nutrient Mixture F-12 Ham (DMEM/F12, Sigma-Aldrich) supplemented with 10% heat-inactivated fetal bovine serum, 2 mM L glutamine, 100 U/mL penicillin, and 100 μg/mL streptomycin (Sigma-Aldrich). HEK-293T cells were incubated at 37 °C in a humidified 5% CO_2_ incubator.

### Cell Transfection and Dual-Luciferase Reporter Assay

At 24 h prior to transfection, the HEK-293T cells were plated at 8 × 10^4^ cells per well in 24-well dishes. Transient transfection was performed at ~ 80% confluence using the FuGENE HD transfection reagent (Promega) according to the manufacturer’s protocol. For each transfection experiment, 500 ng of the appropriate reporter construct (gfap-3′UTR-wt, gfap-3′UTR-mut, or unmodified pmirGLO vector) and 60 nM of MiR124-3p mimic or Negative Control (Dharmacon, USA) were used. For each plasmid, three independent transfection experiments were performed and each was done in quadruplicate. Twenty-four hours after, transfection cells were harvested and assayed for firefly and Renilla luciferase activities by the Dual-Luciferase Reporter Assay System (Promega) in a GloMax-Multi+ Microplate Multimode Reader (Promega), according to the manufacturer’s instruction. Relative luciferase activity was compared using the ratio of firefly and Renilla luciferase activity (F/R) of MiR124-3p to the Negative Control ratio of firefly and Renilla luciferase activity (F/R).

### Cell Viability Assay

To measure cell viability, CellTiter-Glo Luminescent Cell Viability Assay was used (Promega). It measures the viability of cells based upon the quantification of cellular ATP levels. The assay relies upon the generation of a luminescent signal which is proportional to the amount of ATP and thus the number of cells that are present. For each plasmid, one additional transfection experiment was performed in quadruplicate. To obtain a value for background luminescence, control wells containing medium without cells were prepared. After transfection procedure, the volume of CellTiter-Glo Reagent equal to the volume of cell culture medium present in each well was added. Contents were mixed for 2 min to induce cell lysis and incubated at room temperature for 10 min. Luminescence signal was measured in GloMax-Multi+ Microplate Multimode Reader (Promega).

### Fish Handling and Exposure

Hatchery-reared juveniles of whitefish (*Coregonus lavaretus*, 98.8 ± 8.5 g mean weight, 24.0 ± 0.7 cm mean length) were held at the Department of Salmonid Research in Rutki (Inland Fisheries Institute in Olsztyn, Poland). The fish were acclimated for 2 weeks at 10 °C. During the acclimation period, the whitefish were fed four times a day (Skretting). After acclimatization, the fish were deprived of food for 2 days, then anesthetized by immersion in etomidate solution prior to injection. The MC-LR dosage was selected based on our earlier studies in whitefish (Brzuzan et al. 2016; Woźny et al. 2016). Chemical standard of MC-LR (purity ≥ 95%; HPLC) was obtained from Enzo Life Sciences (Enzo Biochem, Inc.) and dissolved in saline solution (0.8% NaCl) as a vehicle solvent. The prepared solution contained 10 μg of MC-LR per 200 μL of volume set for each intraperitoneal injection. Exposed whitefish individuals received single intraperitoneal injections (0.5 × 25 mm needle) of MC-LR (100 μg kg^−1^ body wt.; 10 μg per 200 μL^−1^ of 0.8% NaCl), and in the same way, control fish were injected with an equal volume of saline solution alone. Following injection, the fish were placed in separate single-pass flow-through tanks supplied with system (surface) water for 48 h (pH 7.5; oxygen saturation > 90%; ammonium < 0.134 mg L^−1^). The intraperitoneal injections of MC-LR or saline were repeated on days 7, 14, and 21. Finally, six whitefish sampled immediately prior to placing in flow-through tanks were taken as an initial control (0 h). Water temperature was measured once a day. The 95% confidence interval of the temperature in the tanks with exposed and control fish was 8–9 °C. All animals used in this study were handled in accordance with the regulations set forth by the Local Ethical Commission in Olsztyn (resolution No. 100/2011 issued on 23rd of November 2011).

### Collection of Fish Tissue and Plasma Samples

At the times of collection (0 h and after 1/3 days, 1 days, 2 days, 7 days, 14 days, 28 days), the fishes were anesthetized with etomidate solution and around 2 mL of whole blood was taken from the caudal vein using S-Monovette K3 EDTA (Sarstedt). Then, the fish were killed and the brain was dissected out and preserved in RNAlater (Sigma-Aldrich). After collection, the blood was mixed by gently inverting the tube several times and immediately centrifuged at 4000×*g* at room temperature for 5 min. The plasma layer (approximately 400 μL) from the top of the tube was transferred into a fresh tube. Plasma was stored at − 20 °C during sample collection, shipped on dry ice, and stored at − 80 °C in the laboratory until next procedures.

### Extraction and Reverse Transcription of Total RNA from Brain Tissue

Total RNA was extracted from the midbrain (approximately 20 mg) from control and MC-treated whitefish using a mirVana isolation kit (Life Technologies) according to the manufacturer’s protocol. Briefly, 200 μL of Lysis/Binding Buffer was added. Samples were vortexed vigorously for 1 min. Then, 20 μL of miRNA Homogenate Additive was added and mixed by inverting the tube several times before it was left on ice for 10 min. Next, 200 μL of Acid-Phenol:Chloroform was added and the mixture was vortexed for 1 min. To separate the aqueous and organic phases, the tubes were centrifuged for 5 min at maximum speed (10,000×*g*). About 200 μL of upper phase was then transferred into a fresh tube. Next, 1.25 volumes of 99.8% ethanol at room temperature were added, and the entire solution was transferred into individual filter cartridges before centrifugation for 30 s at 10000×*g*. Initial washing was conducted with 700 μL of Washing Buffer 1, followed by two washes with 500 μL of Washing Buffer 2/3. RNA was eluted from the washed filter cartridges with 40 μL of preheated (95 °C) Elution Solution. The extracted RNA was immediately used in the next step. For mRNA expression studies, total RNA was DNAse treated, according to the manufacturer’s protocol (Turbo DNAse; Ambion; USA). Reverse transcription (RT) was then directly carried out using a RevertAid First Strand cDNA synthesis kit (Thermo Scientific; USA). The cDNA synthesis reaction contained 1 μg of DNAse-treated total RNA and 5 μM of oligo(dT)_18_ primer. After optional incubation (65 °C for 5 min), the samples were chilled on ice and the following components were added: 4 μL of 5× Reaction Buffer, 20 U of RiboLock RNase Inhibitor, 1 mM of dNTP mix, and 200 U of RevertAid M-MuLV Reverse Transcriptase. To check for DNA contamination, two additional control reactions were run, one with all RT components except the enzyme (RT-), and the other with DNAse-treated RNA and no other reagents (NTC). The reaction was carried out at 42 °C for 60 min, and then terminated by heating at 70 °C for 5 min. Synthesized cDNA samples were stored at − 80 °C and thawed only once, just before amplification.

To profile MiR124-3p expression, we designed a protocol based on polyadenylated RNA and stem-loop reverse transcription (Biggar et al. 2014; Brzuzan et al. 2016). miRNA polyadenylation was performed using a polymerase tailing kit (Epicentre). Reactions were prepared with 1 μL of 10× polyadenylate polymerase buffer, 1 μL of adenosine triphosphate (ATP, 10 mM), 0.5 μL of *Escherichia coli* poly(A)polymerase (4 U), 1 μg of total RNA, and RNase-free water to a final volume of 10 μL. Reaction mixtures were incubated at 37 °C for 30 min, followed by 95 °C for 5 min to terminate the adenylation, and then transferred directly to ice. Reverse transcription was performed as described above, with one modification. Instead of using oligo(dT)18 primer, an aliquot of 10 μL of polyadenylated RNA from the previous step was incubated with 1 μL of 100 μM universal stem-loop RT primer (5′-CTC ACA GTA CGT TGG TAT CCT TGT GAT GTT CGA TGC CAT ATT GTA CTG TGA GTT TTT TTT TVN-3′), followed by already described procedure. Synthesized cDNA samples were diluted (20×) stored at − 80 °C and thawed only once, just before amplification.

### miRNA Extraction from Plasma

Total RNA was extracted from 80 μL of plasma from control and MC-treated whitefish using a mirVana isolation kit (Life Technologies) according to the manufacturer’s protocol with modifications. After thawing, samples were briefly centrifuged and 10 volumes of Lysis/Binding Buffer were added. Samples were vortexed vigorously for 1 min. To normalize sample-to-sample variation in the RNA isolation procedure, an exogenous spike-in control of 3 μL of synthetic 5 nM cel-MiR39-3p (5′-UCACCGGGUGUAA AUCAGCUUG-3′) was added. Then, 80 μL of miRNA Homogenate Additive was added and mixed by inverting the tube several times before it was left on ice for 10 min. Next, 800 μL of Acid-Phenol:Chloroform was added and the mixture was vortexed for 1 min. To separate the aqueous and organic phases, the tubes were centrifuged for 5 min at maximum speed (10,000×*g*) at room temperature. About 300 μL of upper phase was then transferred into a fresh tube. Next, 1.25 volumes of 99.8% ethanol at room temperature were added, and the entire solution was transferred into individual filter cartridges before centrifugation for 30 s at 10000×*g*. Initial washing was conducted with 700 μL of Washing Buffer 1, followed by two washes with 500 μL of Washing Buffer 2/3. RNA was eluted from the washed filter cartridges with 40 μL of preheated (95 °C) Elution Solution. The extracted RNA was immediately used in the next steps described above.

### qPCR Analysis

Real-time PCR was used to determine *gfap* mRNA and MiR124-3p levels in midbrain and plasma of control and MC-LR-treated whitefish. Reactions were carried out in final volumes of 20 μL, consisting of 10 μL of Power SYBR Green PCR Master Mix (Life Technologies, USA), 0.25 μM of each primer (forward and reverse; Supplementary File 1), 1 μL of cDNA template (see “Determining *gfap* cDNA Sequence of European Whitefish” and “Phylogenetic Analysis of Whitefish Gfap”), and 7 μL of PCR-grade water. Amplification was performed on an ABI 7500 Real-time PCR System thermocycler (Applied Biosystems; USA) with the following conditions: 95 °C for 10 min, then 45 cycles of 95 °C for 15 s and 60 °C for 1 min. The reaction for each sample was carried out in duplicates. No template controls (NTCs) were included to test for the possibility of cross-contamination. To check the quality of each PCR products, melting curve analyses were additionally performed after each run.

Quantitative cycle (Cq) values obtained from qPCR were converted into template concentration using a standard curve plot (Cq versus log DNA concentration), following the approach of Arukwe (2006) as described by Spachmo and Arukwe (2012). To generate the standard curve, plasmids with target sequences were used to prepare a series of six tenfold dilutions and then served as the template in qPCR. Cq values obtained for each dilution were plotted against the log of the DNA concentration and then used to extrapolate the unknown samples to absolute numbers. In order to calculate relative expression, the absolute numbers of all analyzed samples were divided by the geometric mean obtained from the control group; and these values were further presented as the expression ratio (*R*). Based on the results obtained for the standard curve, PCR efficiency was also calculated according to the following equation: *E* = 10^[−1/slope]^ (Pfaffl et al. 2002).

### Statistical Analysis

The gene (*gfap*; MiR124-3p) expression data obtained from the treatment study was tested for statistical differences using an unpaired *t* test (two-tailed) to compare the values determined in control fish cohort with those of the exposed whitefish at the respective time points. To test for normality of the distribution of the samples compared, the non-parametric Kolmogorov-Smirnov (K-S) test was used. In cases where the K-S test failed, two-sample Mann-Whitney test was applied. The correlation between expression of MiR124-3p in the brain and peripheral blood serum was analyzed using the Pearson method. All calculations and statistical analyses were performed using GenEx 5 Professional software (MultiD Analyses).

## Results

### Gfap in Whitefish Is a Structurally Conserved Protein

**Fig. 1:**
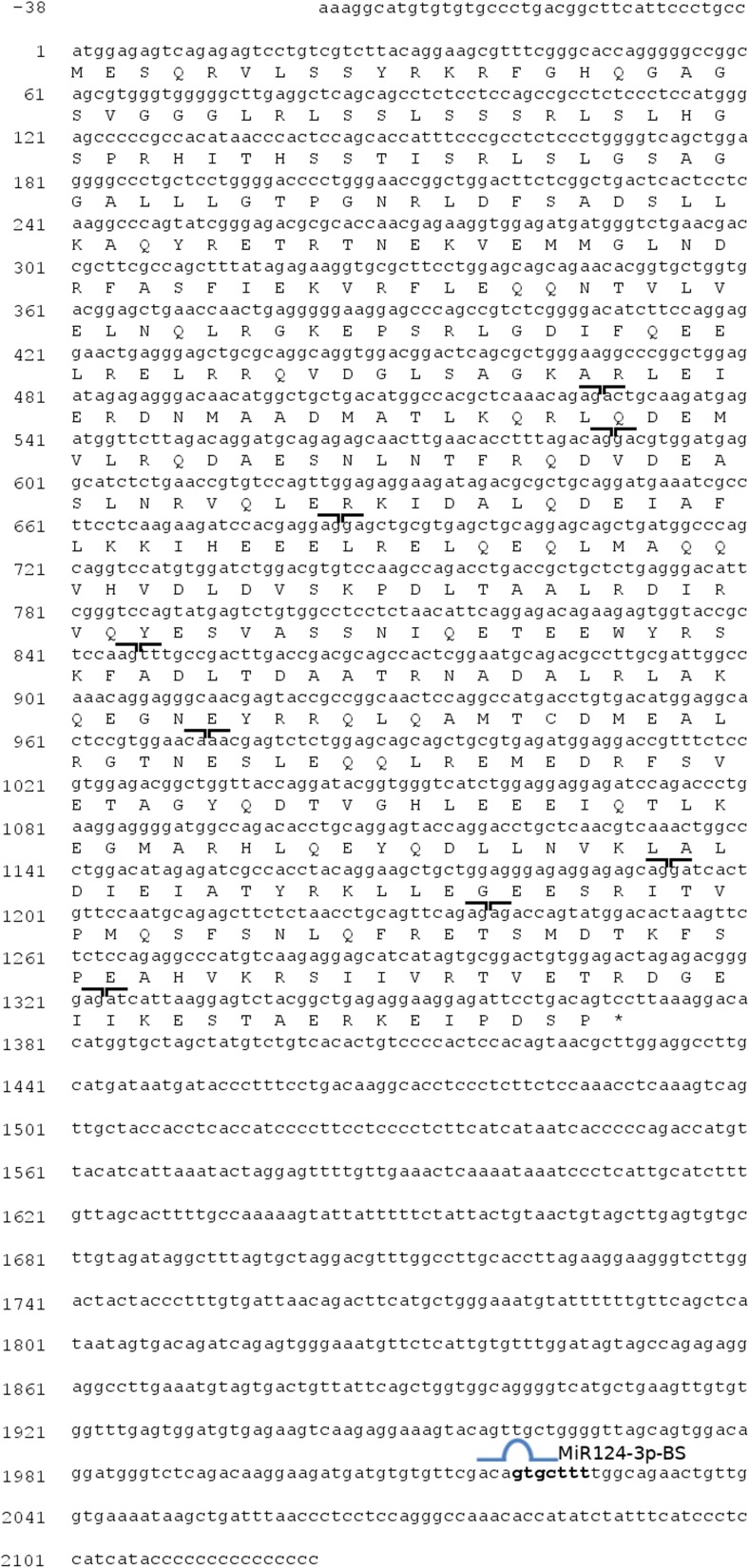
Nucleotide sequence of cDNA of whitefish *gfap* aligned with deduced amino acid sequence (GenBank accession no. MG182670). Predicted exon–exon junctions and predicted MiR124-3p binding site (MiR124-3p-BS) are indicated

**Fig. 2:**
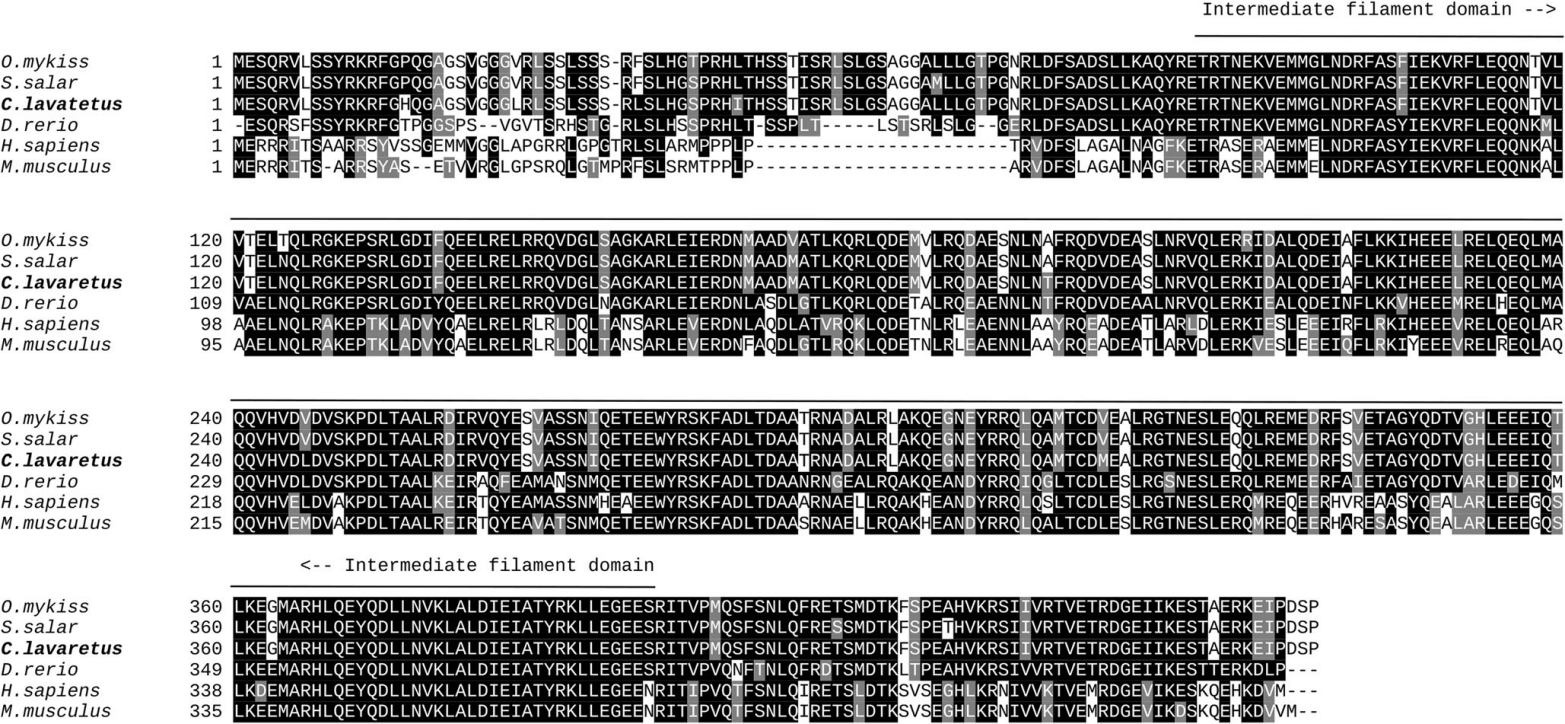
Multiple alignment of deduced amino acid sequence for whitefish Gfap with those for other species. The alignment was built using Clustal W and presented using BoxShade. Identical residues are white letters in black boxes, while conservative substitutions are shaded in gray. The Gfap-conserved functional domains are indicated on the top of the region

The obtained cDNA sequence of whitefish *gfap* comprised 2160 base pairs, including partial 5′UTR (38 bp) and partial 3′UTR (748 bp) (Fig. 1). The cDNA of *gfap* encoded a Kozak consensus sequence gccAUGG (Fig. 1; nucleotides from − 3 to + 4). The open reading frame encodes 457 amino acids long protein. The analysis showed also that the estimated molecular weight of the putative protein is 52.1 kDa with a theoretical isoelectric point of 5.26. The predicted protein contains one putative functional Gfap homology intermediate filament domain: 90–398 aa (*E* value = 1.83e^−126^). The length of the full deduced amino acid sequence of whitefish Gfap (457 aa) differs from the compared species’ aa sequences by 27 (*M. musculus*), 25 (*H. sapiens*), 14 (*D. rerio*), or is the same (*O. mykiss* and *S. salar*). A multiple alignment of the deduced whitefish Gfap protein sequence with those of other species retrieved from GenBank (Fig. 2) allowed the calculation pairwise identity score matrix which demonstrated that the whitefish Gfap protein shared 98% identity with salmon and trout, 81% with zebrafish, and 63% with human and mouse. In addition, the neighbor-joining tree showed that whitefish Gfap clustered together with salmon and trout (100% of bootstrap value) and then coalesced with zebrafish (87%). The second group consisted of human and mouse, which formed another cluster with 100% frequency of occurrence. Together, these data suggest that whitefish Gfap is a structurally conserved protein, which retains functional features characteristic for intermediate filament protein.

### A MiR124-3p Target Site Is Present in the 3′UTR of *gfap* in Whitefish

**Fig. 3:**
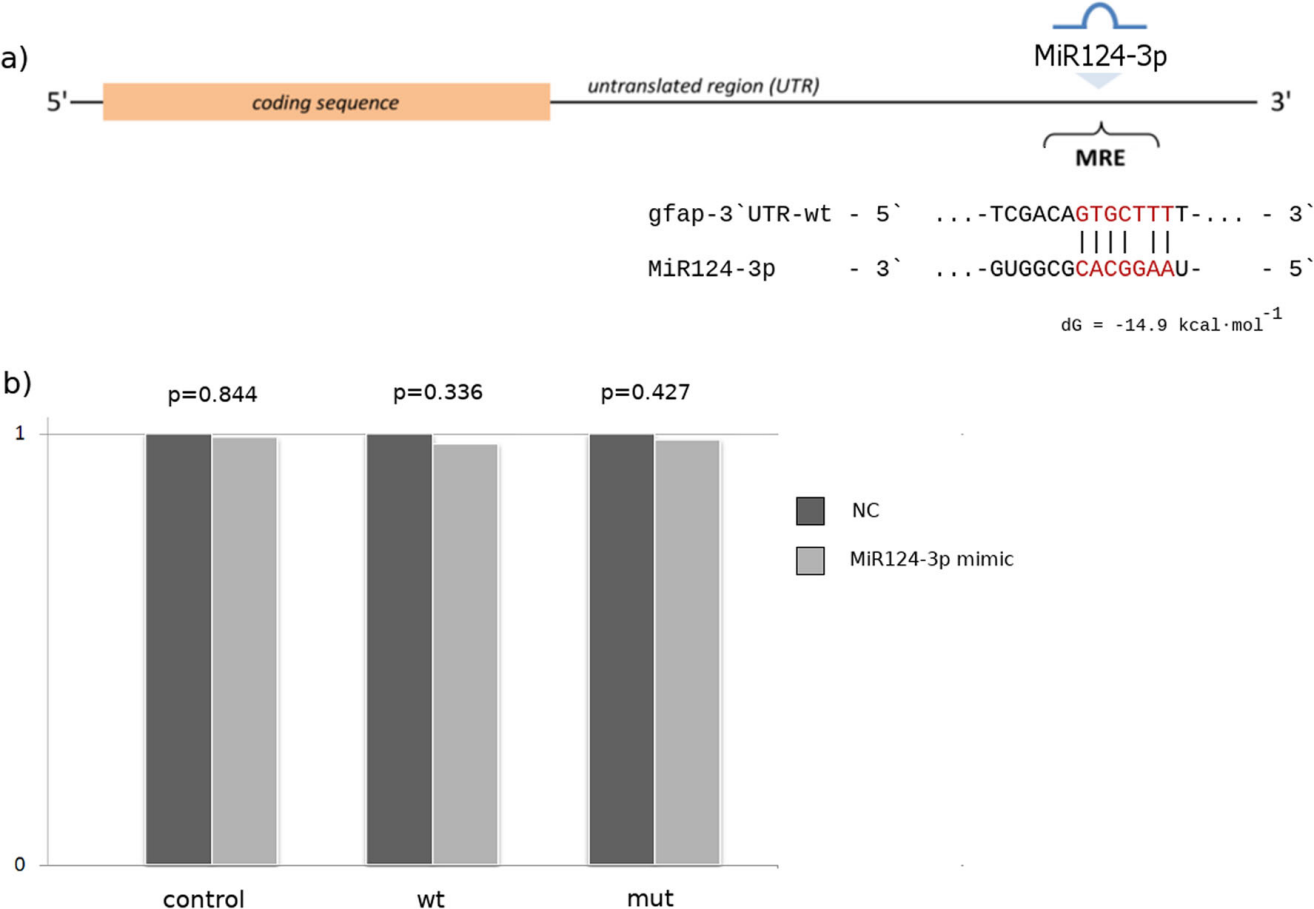
Whitefish gfap 3′UTR is not a functional target of MiR124-3p in vitro. **a** Diagram of the MiR124-3p putative binding sites in the 3′UTR of *gfap*. **b** Effects of MiR124-3p mimic on the expression of gfap 3′-UTR-containing reporter genes (wt, wild type; mut, mutant type). Each luciferase activity was normalized to the value obtained in the cells transfected with NC mimics. Results were represented as mean ± S.D. from three independent experiments, each prepared in quadruplicate

One of the objectives of our study was to investigate whether the 3′UTR of *gfap* in whitefish contains a putative MiR124-3p response element(s). In silico analysis, using Segal Lab software identified one MiR124-3p target site within the analyzed 3′UTR sequence (Fig. 1). The strength of the potential miRNA:mRNA interaction could be estimated in terms of the minimum free energy for hybridization (ΔG), which for the predicted target site equals − 14.9 mol^−1^. Figure 3a shows the putative base-pairing between mature MiR124-3p and its predicted target region within the 3′UTR of whitefish *gfap* mRNA. The degree of complementarity to the seed region (nucleotides 2, 3, 5, 6, and 7 of microRNA) classifies this target site into the so-called *GUT moderate-stringent* seed class, which contains one G:U wobble with the uracil on the target site of mRNA (Saito and Sætrom 2010).

### *gfap* Is Up-regulated from the 7th Day of Exposure to MC-LR

**Fig. 4:**
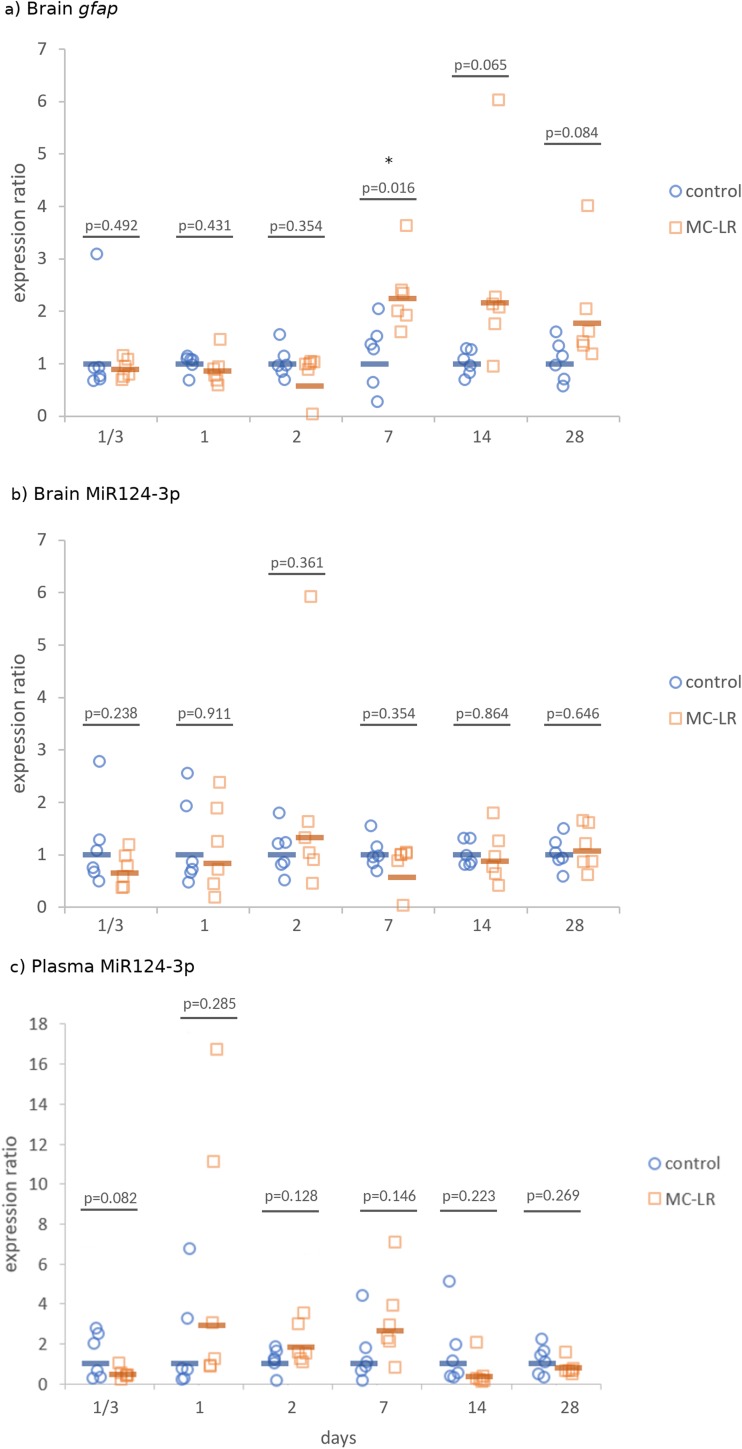
Expression ratio of **a** *gfap* in midbrain, **b** MiR124-3p in midbrain, and **c** MiR124-3p in plasma of whitefish exposed for 28 days to microcystin (MC-LR) at a dose of 100 μg kg^−1^ (orange squares), relative to control (blue circles). Intraperitoneal injections of MC-LR were given on days 0, 7, 14, and 21. Points present values obtained from individual fish within a specific group, whereas horizontal lines indicate mean values (*n* = 6 per group) relative to control at the respective exposure period. Asterisks indicate significant differences from the control group at that time (treatment-dependent changes; *p < 0.05)

Starting on the 7th day of exposure, we observed up-regulation of *gfap* expression relative to the untreated control groups (Fig. 4a). While the differences between gfap expression levels were significant at 7 days after injection (*p* = 0.016), those observed until the last day of experiment were not: 14 days (*p* = 0.065) and 28 days (*p* = 0.084). This up-regulation of *gfap* expression may serve as a defense mechanism against MC-LR.

At the same time, MiR124-3p levels in the whitefish brains remained unchanged (Fig. 4b) so as plasma levels of this miRNA (Fig. 4c), which suggest no functional correlation between *gfap* and MiR124-3p expression levels in MC-LR triggered toxicity in whitefish.

### The 3′UTR of Whitefish *gfap* Is Not a Functional Target of MiR124-3p In Vitro

In order to verify the findings from in vivo experiment, we performed dual-luciferase reporter assay. This is the first study to investigate whether *gfap* is negatively regulated by MiR124-3p. Cotransfection of HEK-293T with the gfap-3′UTR-wt luciferase reporter plasmid and MiR124-3p mimic did not lead to any inhibition of luciferase activity. The same results were observed with gfap-3′UTR-mut construct. These data show that studied g*fap* 3′UTR was not a direct target for MiR124-3p in vitro (Fig. 3b).

## Discussion

This study investigates the possibility of functional correlation between *gfap* and MiR124-3p expression in MC-LR-exposed whitefish, and the suitability of plasma MiR124-3p as a potential biomarker of brain injury. We found that MC-LR induced the expression of *gfap* mRNA from the 7th day of the exposure period and kept it slightly elevated to the end of the treatment. This indicates that MC-LR may induce functional changes in the brain of whitefish that could be caused due to injury of that organ. Although MCs are primarily considered to be hepatotoxins, several studies have demonstrated that MC exposure causes a variety of symptoms related to neurotoxic effects (reviewed in Florczyk et al. 2014). For example, MC-LR exposure produced inflammatory effects in rat brains: astrocyte hyperplasia and increased expression of Gfap protein, which indicated that astrocytes actively respond to the toxicity of MCs (Li et al. 2014). Reactive astrocytes not only secrete inflammatory mediators and chemokines that contribute to inflammation-mediated CNS damage (Meeuwsen et al. 2003) but also produce inflammatory cytokines and chemokines that contribute to reparative processes in the early stages of neuroinflammation (Sharma et al. 2007). Similarly, in the liver of whitefish exposed to MC-LR, numerous macrophages were present on the 7th day of the exposure period, indicating that cell debris from cell damage were being removed and that liver regeneration was beginning (Woźny et al. 2016). Activated macrophages produce cytokines, such as TNF-a, which is mainly involved in the systemic inflammation response, but also in apoptosis, cell proliferation, and cell differentiation (Hehlgans and Pfeffer 2005). Besides inflammation, MC-LR exerts whole brain cytotoxicity (Feurstein et al. 2009), inducing neuronal apoptosis and degrading the neurite network (Feurstein et al. 2011). As shown recently in rats’ astrocytes, cytoskeletal disruption seen by the degradation of GFAP occurred after exposure to various MC variants (Bulc Rozman et al. 2017). Similarly, in the liver of whitefish, cytotoxicity was revealed in disruption of endoplasmic reticulum, chromatin, and cytoskeleton (Woźny et al. 2016). Although the current study does not include ultrastructural images of whitefish brain, it was reasonable to assume, based on the above-mentioned studies, that similar effects could have emerged. The present study demonstrates that repeated intraperitoneal injection of MC-LR induced neuronal toxicity was depicted in elevated *gfap* expression, which could be associated with inflammatory reactions in the midbrain.

Although *gfap* mRNA expression in the brain was elevated at the 7th day of exposure period, MiR124-3p remained unchanged. This suggests no functional correlation between *gfap* and MiR124-3p expression levels in MC-LR triggered toxicity in whitefish. MiR124-3p seems to play a crucial role in brain development as well it is enriched in mature brain tissue. Besides important roles in neuronal regulation, MiR124 is closely associated with the development of some CNS diseases. Abnormal expression of MiR124 has been shown to be implicated in many CNS diseases, like Alzheimer’s disease, brain tumor, cerebral ischemic stroke, and Parkinson’s disease (Sun et al. 2015). In Parkinson’s disease, MiR124-3p prevented various pathological processes including neurotoxicity, neuronal apoptosis, neuroinflammation, and oxidative stress (Geng et al. 2017). MiR124-3p overexpression attenuated neuronal injury (displayed as increased cell viability and superoxide dismutase activity), as well as reduced cell apoptosis, Caspase-3 activity, lactate dehydrogenase activity, inflammatory factors TNF-α and IL-1β levels, and reactive oxygen species generation (Geng et al. 2017). Possible regulation of the glial scar by MiR124 has been reported by Doeppner et al. (2013) and more recently by Hamzei Taj et al. (2016). MiR124 participated in this process, by increasing expression of Arg-1 or TGF-β in microglia and macrophages, which in turn modulated the reactive astrocytes by the secretion of cytokines and chemokines. On the other hand, MiR124 effectively silenced human *GFAP* promoter expression by targeting its 3′UTR (Taschenberger et al. 2017). Moreover, it has been shown that MiR124 overexpression indirectly suppressed the expression of GFAP (Jiao et al. 2017). Our results showed an unchanged expression profile of MiR124-3p during the exposure period, proving that MC-LR-induced changes in *gfap* expression in whitefish is not correlated with participation of this miRNA.

It is accepted that circulating miRNAs are either byproducts of microvesicle secretion or cell death. Extracellular circulating miRNAs are mostly microvesicles free and associated with the RNA-binding Argonaute proteins (Ago), which are thought to be released into the circulation from the cytoplasm of necrotic cells due to disruption of cellular membranes (Sohel 2016). Thus, tissue-specific miRNAs are promising biomarkers of monitoring tissue injury. In this study, plasma levels of MiR124-3p in MC-LR exposed whitefish were unchanged during exposure period which excludes this miRNA as a biomarker of brain injury in fish. Both acetaminophen (APAP) and MC-LR induce acute liver injury in mammals and fish. Drug-induced liver injury can be monitored through traditional blood enzymes (ALT, AST) or more sensitively through miRNA quantification. Our previous study on whitefish exposed to MC-LR showed increased liver-specific miRNA plasma levels in fish, which is similar to the outcome acetaminophen induced in mammals (Florczyk et al. 2016). MiR122-5p levels were elevated as early as 8 h after exposure. This following study aimed to examine if MC-LR-induced brain injury could also be monitored using brain-specific MiR124-3p. Recent study on evaluating MiR124 as a marker of acetaminophen-induced brain injury in pigs demonstrated increased levels of this miRNA in blood (Baker et al. 2015). However, the release of MiR124 into the plasma was likely to be due to relative ischemia, as increased levels of MiR124 were significantly elevated only when cerebral perfusion pressure (CPP) fell below 30 mmHg. This could be related to indirect acetaminophen-induced brain injury caused by ischemia and general organ failure (Baker et al. 2015). In the present study, repeated exposure of whitefish to a subacute dose of MC-LR could not induce similar changes.

Luciferase reporter assay confirmed the lack of interaction in vitro which shows that MiR124-3p is unlikely to regulate *gfap* expression. This is the first study to investigate whether whitefish *gfap* is negatively regulated by MiR124-3p; however, our prediction of potential MiR124-3p target sites in 3′UTR of *gfap* could have been not accurate. For our analysis, we used miRNA prediction tool developed by Segal Lab, which has found only one moderate-stringent seed class MiR124-3p binding site. This miRNA seed class is considered as a weaker binding site than most commonly used in reporter assays 7-mer-m8 seed class. Therefore for our in vitro analysis, with only one binding site present, we have used the whole whitefish gfap 3′UTR sequence.

This prevented any false negative results even if our prediction was not completely correct and stronger MiR124-3p binding sites are present in *gfap* 3′UTR.

In conclusion, our study demonstrates that the exposure of whitefish to MC-LR-induced expression of *gfap* mRNA but did not involve any changes in expression of MiR124-3p in whitefish brain, contributing to our understanding of the mechanistic role of miRNAs in MC-LR neurotoxicity. In addition, we show that the expression of MiR124-3p in whitefish brain and plasma is not correlated with the changes of *gfap* mRNA expression and thus cannot be considered as a biomarker of brain injury induced by MC-LR. Furthermore, by using luciferase reporter assay, we confirmed the lack of interaction between 3′UTR of *gfap* mRNA and MiR124-3p, which rules out the possibility of their direct regulatory relationship. Further research focusing on regulation of astrocyte reactivity could provide better understanding of molecular background underlying MC-LR-induced neurotoxicity and perhaps hold promise for prevention strategy for MC-LR intoxication.

## Electronic Supplementary Material

ESM 1 (PDF 26 kb)
