# Supplementary material for: Microcystin-LR-Triggered Neuronal Toxicity in Whitefish Does Not Involve MiR124-3p
Source: Neurotox Res. 2018 Jun 7;35(1):29–40. doi: 10.1007/s12640-018-9920-4 (PMC6313356; doi:10.1007/s12640-018-9920-4)
Supplement: Supplementary file 1 — (PDF 26 kb) [file 12640_2018_9920_MOESM1_ESM.pdf]

Supplementary File 1 Details of the oligonucleotides used in this study

Application	Name		Sequence	Annealing temperature	Product length
Obtainment of gfap cDNA sequence	Cla-gfap-F1	forward	GCCACGCTCAAACAGAGACT	60	134
	Cla-gfap-R1	reverse	CAGCGCGTCTATCTTCCTCT		
	Cla-gfap-2-F2	forward	ACCAGGATACGGTGGGTCAT	58	661
	Cla-gfap-2-R2	reverse	GCCTATCTACAAGCACACTCAA		
	Cla-gfap-2-F3	forward	TGCGGACTGTGGAGACTAGA	58	789
	Cla-gfap-2-R3	reverse	ATATGGTGTTTGGCCCTGGA		
	Cla-gfap-2-F4	forward	GACGGAGCTGAACCAACTGA	56	457
	Cla-gfap-2-R4	reverse	GAATGTTCGAGGAGGCCACA		
Luciferase reporter plasmids preparation	gfap-3’UTR-F-DraI	forward	ACTTTTTAAATGAGAGGAAGGAGATTCCTG	60	779
	gfap-3’UTR-R-XbaI	reverse	TTGCTCTAGAATATGGTGTTTGGCCCTGGA		
	gfap-MRE1-MUT-T	top	TGATGTGTGTTCGACAGTGGTTTTGGCAGAACTGTTG GTG	60	20
	gfap-MRE1-MUT-B	bottom	CACCAACAGTTCTGCCAAAACCACTGTCGAACACACA TCA		
Reverse transcription for quantification of MiR124-3p level	Universal long stem-loop RT		CTCACAGTACGTTGGTATCCTTGTGATGTTCGATGCCA TATTGTACTGTGAGTTTTTTTTTVN	60	-
qPCR	Cla-gfap-RT-F1	forward	GCCACGCTCAAACAGAGACT	60	134
	Cla-gfap-RT-F2	reverse	CAGCGCGTCTATCTTCCTCT		
	MiR124-3p specific	forward	ACACTCCAGCTGGGTAAGGCACGCGGTGAATG	60	95
	Universal primer	reverse	CTCACAGTACGTTGGTATCCTTGTG		
